# Medication Exposures and Subsequent Development of Ewing Sarcoma: A Review of FDA Adverse Event Reports

**DOI:** 10.1155/2015/948159

**Published:** 2015-05-07

**Authors:** Judith U. Cope, Gregory H. Reaman, Joseph M. Tonning

**Affiliations:** ^1^Office of Pediatric Therapeutics, Office of Special Medical Programs, Office of the Commissioner/Food and Drug Administration, Building 32, Room 5156, 10903 New Hampshire Avenue, Silver Spring, MD 20993, USA; ^2^Office of Hematology and Oncology Products, OND, Center for Drug Evaluation and Research, U.S. Food and Drug Administration, White Oak Bldg. 22, Room 2202, 10903 New Hampshire Avenue, Silver Spring, MD 20993, USA; ^3^U.S. Public Health Service Commissioned Corps, Office of Translational Sciences, Center for Drug Evaluation and Research, U.S. Food and Drug Administration, White Oak Bldg. 22, Room 3410, 10903 New Hampshire Avenue, Silver Spring, MD 20993, USA

## Abstract

*Background*. Ewing sarcoma family of tumors (ESFT) are rare but deadly cancers of unknown etiology. Few risk factors have been identified. This study was undertaken to ascertain any possible association between exposure to therapeutic drugs and ESFT. *Methods*. This is a retrospective, descriptive study. A query of the FDA Adverse Event Reporting System (FAERS) was conducted for all reports of ESFT, January 1, 1998, through December 31, 2013. Report narratives were individually reviewed for patient characteristics, underlying conditions and drug exposures. *Results*. Over 16 years, 134 ESFT reports were identified, including 25 cases of ESFT following therapeutic drugs and biologics including immunosuppressive agents and hormones. Many cases were confounded by concomitant medications and other therapies. *Conclusions*. This study provides a closer look at medication use and underlying disorders in patients who later developed ESFT. While this study was not designed to demonstrate any clear causative association between ESFT and prior use of a single product or drug class, many drugs were used to treat immune-related disease and growth or hormonal disturbances. Further studies may be warranted to better understand possible immune or neuroendocrine abnormalities or exposure to specific classes of drugs that may predispose to the later development of ESFT.

## 1. Introduction and Study Objective

Ewing sarcoma family of tumors (ESFT) is a group of small, round blue cell tumors that arise in the bone or soft tissue. ESFT include classic Ewing sarcoma (ES) of bone, extraskeletal ES, skin tumors of the chest wall, and primitive neuroectodermal tumors (PNET) of the bone or soft tissue. About 90% of ESFT cases are characterized by the chromosome translocation t(11;22)(q24;q12) which involves the EWS/FLI-1 fusion gene and may share a common neural histogenesis [1–4]. While ESFT is rare, it is the second most common primary malignant bone tumor occurring in children and young adults and accounts for 10–15% of all primary bone tumors and 3% of all pediatric malignancies. ESFT occurs with a male predominance and highest incidence rates among whites with considerably lower rates in Blacks and East Asian populations. Age of onset is normally in the second decade of life, with 80% occurring in the first 2 decades of life and 80% occur in the skeleton [2–5]. Each year up to 400 patients in the US will be diagnosed with ESFT. The incidence has been steady over the past few decades [6].

While few risk factors for ESFT have been identified, the racial difference and specific chromosomal translocation might suggest a genetic predisposition [1, 7]. However, unlike other bone cancers (e.g., osteosarcoma) and soft tissue sarcomas (e.g., rhabdomyosarcoma) ESFT has not been found to be associated with any genetic disease or hereditary cancer syndrome [2, 7, 8]. Epidemiological studies have examined a wide array of possible risk factors, including childhood conditions and various parental exposures [9–16]. There is limited data about drug exposures as possible risk factors for ESFT. One study reported a possible association with poison or overdose of medications [11]. Another study by Valery et al. 2003 [16] found medication use to be more prevalent in controls than cases. While few if any studies have found an association with medications and ESFT (with exposure to medications), two studies [10, 16] found an inverse association with asthma, but this has not been confirmed by the other studies [9, 11, 12, 16]. Despite continuing efforts to identify risk factors for ESFT, its etiology is still unknown.

The aim of this study was to review all cases of ESFT that have been reported to FDA from January 1, 1998, through December 31, 2013, and to assess any possible association with therapeutic products. (FAERS includes therapeutic agents which include drugs and also biologics.) We also conducted an extensive literature search of articles that pertained to the epidemiology of ESFT.

## 2. Methods 

All case reports for drug products that included ESFT from the FDA Adverse Event Reporting System (FAERS) database were analyzed. The FAERS database, including foreign and domestic reports and all age groups, was searched for the time period, January 1, 1998, through December 31, 2013. The following Medical Dictionary for Regulatory Activities (MedDRA) search terms (MedDRA website, http://www.meddra.org/ see [17]) were used to identify any reports of cases with ESFT: Ewing's sarcoma, Ewing's sarcoma metastatic, Ewing's sarcoma recurrent, extraosseous Ewing's sarcoma, extraosseous Ewing's sarcoma metastatic, extraosseous Ewing's sarcoma nonmetastatic, extraosseous Ewing's sarcoma recurrent, neuroectodermal neoplasm, primitive neuroectodermal tumour, primitive neuroectodermal tumour metastatic, and peripheral primitive neuroectodermal tumour of soft tissue. Cases were stratified by year of occurrence and defined by the reporter as definite, probable, possible, and unlikely in terms of the diagnosis of ES and potential association with drug exposure. Reports of ESFT as a secondary malignancy, as a primary tumor preceding a secondary malignancy, adverse events related to chemotherapy, and other treatments for ESFT and report duplications were excluded. Case reports of medication use pre-ESFT were analyzed by two independent reviewers.

*Data Source*. This study utilized the FAERS database of adverse event (AE) reports. Since 1969, FDA has maintained this passive surveillance system to detect problems with drugs and biological products used by humans. Manufacturers are required by US law to report AEs associated with their products, and AEs may also be spontaneously reported by health care professionals and consumers. The database contains reports from the US and non-US countries. Reports that the reporter thought to be related to the use of a product are reviewed, coded, and accumulated into an electronic database. The adverse events are codified by MedDRA terminology. This passive surveillance program functions as an early warning system for the detection of serious AEs not identified during the premarket testing or clinical trials. A special feature of this program includes follow-up reports of cases following FDA's request for further information. Some of the reports in this database may also be found in the published literature.

## 3. Results

### 3.1. Overall FAERS Reports Summary

Over the 16-year time period, 134 reports were retrieved with mention of ESFT. After clinical review of the narrative reports 25 cases were identified with a history of medication use prior to ESFT diagnosis, most with a history of ≥18 months. Age range was 5–68 years of age (median 24 years) (Table 1). Many of our cases were outside the adolescent age group. 

*Primary ESFT after Drug Use (n* = 25*) (Table 2)*. A total of 25 out of 134 reports revealed the use of various suspect drug products with subsequent development of ESFT. Reports were excluded if ESFT occurred as a secondary malignancy, drug adverse events occurred with ESFT therapy, or ESFT occurred as a primary cancer before a secondary cancer occurred that followed drug therapies. The 25 cases of ESFT ranged in age from 4 to 68 years (median 24 years). There was no age information for 4 cases. Thirteen were US cases. Latency to ESFT diagnosis was ≥1 year for 14 cases, <1 year for 5, and unknown for 4. The reported medications included immunosuppressive drugs or drugs used for immune-related disorders (e.g., interferon beta-1, peginterferon alpha-2A and ribavirin, tumor necrosis factor (TNF) blockers, methotrexate, cyclosporine, and azathioprine), isotretinoin, recombinant growth hormone (rGH), and psychotropic medications including antiepileptics (AEDs), atypical antipsychotics, and psychostimulants. Many of the incident cases were confounded by concomitant medications that are labeled for possible carcinogenicity risk.

Twelve cases were treated with immunosuppressive products for a variety of conditions including 3 Crohn's, 3 multiple sclerosis (MS), 1 renal transplant, 1 rheumatoid arthritis (RA) and 1 each of ankylosing spondylitis, psoriasis vulgaris, chronic hepatitis C genotype 1b, and nephrotic syndrome. Use of more than one immunosuppressant drug was mentioned in 6 of the 12 reports.

Three cases received growth hormone (GH) and were diagnosed with ESFT 18 months to 4 years after beginning therapy. One child was treated with somatropin for microsomia due to neurosecretory GH disorder and a second child was treated for GH deficiency. The third case was a patient with Crohn's disease who received immunosuppressants and GH (this case is included in the 12 immunosuppressant cases above).

Three cases received other hormonal products, including parathyroid hormone, combined estrogen/progesterone, and etonogestrel implant. The one case treated with recombinant human parathyroid hormone had failed several earlier therapies for osteoporosis. The one case taking combined estrogen/medroxyprogesterone was a menopausal woman who was diagnosed with ESFT 10 months later, and the third case with the etonogestrel implant was a morbidly obese young woman (268 pounds, body mass index (BMI) = 42).

Six cases were treated with psychotropic medications, including 2 with atypical antipsychotics (1 olanzapine for unspecified condition, 1 olanzapine and antiepileptic drugs (AED), clonazepam, for depression), 1 attention deficit hyperactivity disorder (ADHD) drug, methylphenidate, 1 case taking 3 AEDs (ethosuximide, valproic acid, and clobazam), 1 case treated with sodium oxylate for narcolepsy, and 1 case treated with another AED, pregabalin, for neuropathic pain.

There were 2 additional cases that received other medications and then developed ESFT, including 1 young adult treated with isotretinoin for acne and developed ESFT 9 months later and 1 young child treated with atorvastatin for familial hypercholesterolemia.

## 4. Discussion 

The FAERS is a passive surveillance system that collects data on adverse events possibly related to drugs and biologics that are reported to FDA by manufacturers, user facilities, and voluntary reporters, such as health care professionals and consumers. Importantly this system may capture rare and serious events that may not be detected in clinical trials and other studies and may lead to further understanding of possible associations of drugs and other risk factors for various diseases.

While few studies have found any association of exposure to medications with ESFT, our retrospective review of FAERS reports identified 25 cases of ES that followed drug therapy for various conditions. The majority were immunosuppressive agents used to treat autoimmune disorders. Most of the other reported drugs included hormones and psychotropic medications. We also reviewed the published literature to see if our findings might be consistent with other studies and case reports.

While various other malignancies have been reported in association with each of the drugs reported in the FAERS cases, there have been few literature reports of ESFT with the use of these medications. Many of the immunosuppressant drugs and some of the psychotropic and hormonal products are labeled with warnings of increased risk for malignancy or have product labeling for carcinogenicity based on animal studies (Table 3).

While the possible carcinogenic effects would differ for all these products, it may be important that most of the drugs in our case series may affect the hypothalamus and pituitary axis causing growth and neuroendocrine disturbance.

### 4.1. ESFT Cases following Immunosuppressive Agents

Nine of the 25 (36%) ESFT cases had been previously treated with immunosuppressive agents for autoimmune disorders including Crohn's disease, MS, RA, psoriasis, and ankylosing spondylitis. Three other cases receiving immunosuppressive therapy included 1 each for renal transplant, chronic hepatitis C, and nephrotic syndrome. Most were older individuals who had received immunosuppressive products two years or more prior to developing ESFT. While many of the case reports did not provide detailed information about duration of therapy and dosage, several reports indicated the use of combination or multimodal therapy with a variety of agents including cytotoxic drugs (e.g., cyclophosphamide, azathioprine, and methotrexate), antibodies and other drug and biologic products for example, interferons, TNF blockers (infliximab, etanercept, and adalimumab), also mesalamine (aminosalicylate, an anti-inflammatory), and 1 case with glucocorticoids.

While ESFT per se has not been reported in association with immunosuppressant drugs, increased exposure to immunosuppressive agents has been found to be related to other cancers [21, 22]. In particular patients receiving higher potency and dosing of therapies for organ transplants have increased cancer risk of non-Hodgkin's lymphoma (NHL), Kaposi's sarcoma, nonmelanoma skin cancer, and other malignancies [23, 24]. While the literature highlights the accumulating evidence for elevated cancer risks with some of the immunosuppressant drugs, further studies are needed especially for the newer biologic treatments for immune-mediated inflammatory diseases. It is unclear whether there may be an elevated risk of extraintestinal solid cancers with the newer biologic agents including TNF blockers [25]. There are several limitations in the studies evaluating cancer risk among patients with exposure to immunosuppressive drugs. While no increase in risk of malignancies overall or in cancer subgroups may be found in clinical trials, study patients may not be a representative sample population and the length of follow-up might not be sufficiently long for malignancies to occur [26]. It is not always clear if it is the underlying autoimmune condition, the immunosuppressive therapy (a specific agent, dosage, or number of therapies) [27], or even potentially oncogenic viruses that play a major role in causing these cancers [28]. Several studies have found consistent increased cancer risk for patients with various underlying inflammatory immune diseases, including Crohn's and RA [29–32]. While the overall cancer incidence and mortality risk are similar to the general population in inflammatory bowel disease, there is an increased cancer risk for Crohn's and slightly increased risk for RA and psoriasis [21]. Studies have been less consistent in showing increased cancer risk associated with MS [33–35] and ankylosing spondylitis. For ankylosing spondylitis increased cancer risk seems to be associated with radiotherapy [36, 37]. Particularly with newer biologic agents including TNF blockers, further studies are needed to better determine if there is an increased cancer risk with the therapies themselves, with the higher doses, and longer treatments, or whether any increased cancer risk is due to underlying severe disease.

In our case series, most reports had incomplete information with regard to dosage and what additional past therapies had been given for the underlying diseases. While most cases had latency periods over a year, 2 of the MS cases had very short latencies of 3 and 10 months. It seems likely that these may have been misdiagnoses for what was later identified to be ESFT. With ESFT, compared to other malignancies, there is frequently a long period of time after initial symptoms until a diagnosis is made. Studies have shown the mean time to be 19–37 weeks [38–40]. One of the FAERS cases had a history of fibrous dysplasia of the clavicle and then later developed ES at the clavicular site. ES has been reported to mimic fibrous dysplasia [41–43].

From a regulatory perspective, FDA classifies carcinogenic risk of drugs and biologics based on their mechanism of action and hazard identification from in vivo and in vitro nonclinical assays [43]. In our series of 25 FAERS cases, 8 had received immunosuppressive products which are labeled with Box Warnings for carcinogenicity and the other 4 reports included immunosuppressants interferon beta-1a (2 FAERS reports), peginterferon alfa-2b (1 FAERS report), and natalizumab (1 FAERS report) which are not labeled for carcinogenicity. Three of these have not undergone carcinogenicity testing. Despite the clear association between immunosuppressant drugs and increased risk of cancer, many products may fail to predict an increased risk in the preclinical studies [44]. Identification of cancer risk with postmarketing pharmacovigilance is crucial. With regard to ESFT, not only is it a rare malignancy, but unlike other bone cancers such as osteosarcoma, there is no animal model.

We also conducted an extensive literature search for additional cases of ESFT reported with underlying autoimmune disorders or immunosuppressive therapy. In addition to the one renal transplant case in our series which was a case report by Balakrishnan et al. [18], we found three additional ESFT cases following renal transplant in the literature, 1 in a 34-year-old female with vaginal PNET [45], a 15-year-old female with PNET of uterus 9 years after renal transplant for end stage renal disease [46], and a case of unknown age with ESFT of the sacrum following renal transplant [47]. A higher risk of developing malignancy in transplant recipients has been reported [48]. No additional published cases of ESFT associated with other autoimmune disorders or immunosuppressant agents were found.

### 4.2. ESFT Cases following Hormonal Drugs

There were three reports of ESFT following GH therapy, including two with GH deficiency and an additional case with Crohn's disease who received both GH and immunosuppressants (included in the 12 immunosuppressant drug reports above). It remains unclear if GH is a carcinogen. Studies have conflicting results regarding any association with GH and cancer. A French population-based cohort study, The French Safety and Appropriateness of Growth Hormone treatments in Europe (SAGhE), of patients treated with recombinant GH reported an increased risk of bone cancers (*n* = 3 cases observed versus 0.6 expected; SMR 5.00; 95% CI, 1.01–14.63), both osteosarcoma (2 cases, statistically nonsignificant) and Ewing sarcoma (1 case, statistically significant) [49]. The selected population for the study included low risk patients who were treated for conditions of idiopathic isolated GH deficiency, idiopathic short stature, short stature in children born short for gestational age, or isolated GH deficiency associated with a minor craniofacial malformation, such as cleft lip. Patients in the middle risk category with pediatric syndromes such as Turner and Noonan syndrome were excluded. The authors of the study thought the elevated bone cancer risk was biologically plausible as bone cancers occur during rapid bone growth of puberty that relate to the insulin-like growth factor 1 (IGF-1) system. It is known that GH and IGF-I do have mitogenic and antiapoptotic activity and that there is a theoretical risk that GH treatment may be associated with cancer [50]. However, interpretation of the study results is limited by the select patient population, the relatively short-term follow-up, and lack of information on the administered GH doses. Other subsequent studies did not demonstrate any increased cancer risks [51–56]. Currently, while GH does not appear to increase the risk for new malignancy in children without known risk factors, an increased risk of a second neoplasm, mostly intracranial tumors in those with prior radiation, has been reported in childhood cancer survivors treated with GH compared with those not treated [51, 55, 57] and this is in the labeling for GH products. Overall, there remain concerns about the possibility of delayed posttreatment effects of heightened GH and IGF-1 on cancer risk. It is unknown if GH treatment may increase the risk of cancer in patients with short stature. Further ongoing surveillance and longer-term studies are needed.

ESFT is thought to be of neural origin and while neurogenic cancers such as neuroblastoma have been reported after GH, we could find no additional literature reports of ESFT following GH. In our ESFT cases one might wonder about the possible risk factor for cancer: is it the GH or is it related to the underlying growth disorder itself?

The other therapeutic hormonal drugs prior to ESFT diagnosis in our series included progestin, combined estrogen/progestin, and parathyroid hormone in nonadolescent females. Our literature review revealed one case report of ES in a young woman four months following abortion [58]. Her only reported drug exposure was four years of oral contraceptive (levonorgestrel 0.05 mg and ethinylestradiol 0.03 mg). (Pregnancy itself leads to decreased circulating GH and slightly increased IGF.)

### 4.3. Growth and Neuroendocrine Effects of the Drugs Used Pre-ESFT

It is of interest that 16 of the 25 FAERS reports involved various drugs that all have an influence or effect on the hypothalamic-pituitary axis. In particular interferons may be associated growth suppression. Some studies showed that immunosuppressive agents, such as TNF blockers (e.g., infliximab), can also affect the hypothalamic-pituitary-adrenal axis [59]. One of the cases treated with a TNF blocker had also received growth hormone. Many of the psychotropic drugs cause hyperprolactinemia. Antipsychotic drugs may be prone to causing hyperprolactinemia [60] and patients treated with psychostimulants may experience slowing of growth or growth retardation. Small transient decreases in serum IGF-1 may occur early in the treatment of patients with methylphenidate. This is less common in females [61]. Seizure medications, especially valproic acid and ethosuximide, have been known to be associated with hyperandrogenism, menstrual disorders, and polycystic ovary syndrome [62]. Isotretinoin has an effect on the pituitary adrenal axis with mild suppression of pituitary hormones. While long-term studies are needed, Karadag et al. [63] found that isotretinoin appeared to have a negative influence on the GH/IGF-1 axis, with a significant decrease in IGF-1 and IGFBP3 levels after 3 months of isotretinoin treatment. Also, steroids used to treat Crohn's may cause suppression of the HPA axis.

Epilepsy itself [62] has sex-specific effects on hormone levels and reproductive function. Women with seizures have hypothalamic disorders, amenorrhea and reproductive/endocrine disorders, and ovarian cysts [64, 65].

### 4.4. ESFT Cases following Psychotropic Drugs

The other category of pre-ESFT therapies included psychotropic drugs. There was only 1 case of each therapy in this category. Currently, most antipsychotic drugs are not considered to increase the risk of cancer [66]. One product, clozapine, has a separate status given that this molecule shows antiproliferative effects implied in agranulocytosis as well as a potential increased risk for leukemia [67].

### 4.5. Literature Review of Studies and Case Reports of ESFT

Few studies have closely examined drug exposures in children who later develop ESFT. Past registry, case-control studies and case series that collected data on medication use [9, 10, 12, 16] have not consistently identified any specific drug therapy as a risk factor for ESFT (see Table 4). Due to the rarity of ESFT, many studies examining possible risk factors lack the statistical power to definitely prove or disprove any association with medication exposures, underlying diseases or genetic disorders. Several studies and literature reports have found a wide array of different medical conditions, anomalies, and even genetic syndromes in patients who developed ESFT (Table 4). More than one study reported congenital genitourinary and renal anomalies (e.g., cryptorchidism, duplication of ureters and collecting system, and inguinal hernias) and musculoskeletal and spinal anomalies (e.g., cervical ribs, spina bifida, and myelomeningocele) [9, 12–15, 20, 68, 69]. It is of interest that many of these conditions are known to be associated with growth and neuroendocrine dysfunction. Our literature review found reports of ESFT in patients with other genetic syndromes including 4 articles with 5 cases with Down syndrome [20, 70–72], 1 case with chromosome 18q-deletion syndrome [73], and 2 cases with osteogenesis imperfecta [14]. While these syndromes are not uncommon and it might be expected that ESFT would occur, it is of interest that all these syndromes are known to be associated with short stature with abnormal pubertal growth and GH deficiency or pituitary dysfunction. Unlike osteosarcoma there has been no consistent association with tall stature or pubertal bone growth in patients with ESFT [10, 16, 19, 74]. Perhaps it is the hormonal changes and interference with growth aspects of puberty that are awry in the development of ESFT.

While our FAERS reports were submitted to FDA in association with drug use, the reported therapies were used to treat autoimmune disorders and childhood conditions that, especially if severe, might lead to growth and/or endocrine disturbance. Crohn's disease, renal failure (especially posttransplant), psychoses, and epilepsy [62, 64] may all cause or be associated with hyperprolactinemia, growth disturbance, and/or pituitary dysfunction during childhood and adolescence. Thus, these cases might also support that there is an underlying endocrine disturbance in patients with ESFT. Also, the various inflammatory conditions that were treated with immunosuppressants in our FAERS case series may also affect bone health (e.g., RA, Crohn's, and MS) and alter pubertal development. Mood disorders such as anxiety, bipolar disorder, insomnia, and fibromyalgia also involve the HPA axis and GH deficiency is associated with high cholesterolemia, osteoporosis, and short stature.

### 4.6. Limitations

The observation of a small number of cases of an uncommon cancer occurring in association with certain exposures does not provide evidence of any causal link. This study using the FDA database has several limitations including reporting biases for cases from manufacturers as well as health care professionals and consumers. FAERS cases may be reported to FDA from postmarket surveillance studies with incomplete enrollment and reporting is dependent upon physician investigator compliance with submitting forms consistently and accurately, and whether the reported disease is related or unrelated to the therapy is subjective. Submitted reports may lack important information on the underlying disease, comorbid conditions, and prior therapies. These reports do not provide histopathologic confirmation of the cancer diagnosis. Another limitation of this study is that it is a retrospective analysis. However, epidemiological studies on patient populations also have limited value because of the long latency period for most cancers and because most studies lack sensitivity. Most studies approach bone cancers as a group without breakdown into subtypes of osteosarcoma and ESFT which are thought to have very different etiologies. Nonetheless, FAERS is the largest postmarketing drug safety database in the world maintained by a single country, containing more than 9 million reports of “drug-related” (but not necessarily causal) adverse event reports since 1969. Because of its potential comprehensiveness in capturing rare drug-associated adverse events from all over the world, the FAERS data may be useful in generating hypotheses relating to possible causes of a specific rare cancer, which later if warranted may be tested in formal and more rigorous epidemiological studies.

## 5. Conclusions

ESFT is known for its striking unimodal incident peak during puberty and few risk factors have been identified as to its etiology. In this present study we found ESFT cases following the use of immunosuppressant agents as well as hormonal medications. These medications as well as the reported underlying immune disorders and neuroendocrine conditions are associated with abnormal growth and hormonal disturbance. These findings may provide insight as to why this cancer has a peak incidence during adolescence. Perhaps the neuroendocrine abnormalities might even be linked to a common gene. Formal epidemiological or clinical studies should be conducted to further evaluate any possible role of neuroendocrine disturbances with these underlying conditions and drugs in the development of ESFT.

## Figures and Tables

**(a):** 

Report year	Number of cases
1997–1999	1
2000–2004	6
2005–2009	9
2010–2013	9

**(b):** 

Drug use	Number of cases	Patient age (yrs)	Latency
Immunosuppressive^∗^	12	5–68	Mean 4.2 years, median 4 years
Growth hormone (GH)	2^∗^	11, 16	4 months after 5 years therapy, 18 months
Estrogen/progestin	2	24, 47	10 months, 2.5 years
CNS depressant	2	34, 42	22 months, unknown
Atypical antipsychotic	1	17	No information
Antiepileptic	1	8	8 years
Antipsychotic and AED	1	19	3 months
ADHD	1	8	No information
Isotretinoin	1	20	10 months
Statin for hyperlipidemia	1	9	>2 years
rPTH^∗∗^	1	49	1 year

^∗^1 Crohn's patient also received GH.

^∗∗^rPTH, recombinant parathyroid hormone.

**(c):** 

Underlying conditions		
12	Immune-related	3 Crohn's disease, 3 multiple sclerosis, 1 renal transplant, 1 rheumatoid arthritis, 1 nephrotic syndrome, 1 ankylosing spondylitis, 1 psoriasis vulgaris, and 1 chronic hepatitis B

5	Endocrine	2 growth disorders, 1 menopause, 1 osteoporosis, and 1 birth control (patient morbidly obese but no underlying condition reported)

3	Neurologic	1 each of narcolepsy, seizure disorder, and fibromyalgia with sciatica/neuropathic pain

3	Psychiatric	1 each of unspecified depression, psychoses, and ADHD

2	Other	1 each of familial hypercholesterolemia, acne

**Table 2:** Characteristics of 25 FDA case reports of pre-ESFT drug exposures.

	Age/sex	Disease	latency	Site	Drug	Type	Concomitant meds	Other
1	13 f	Crohn's	>2 yrs	Pelvis	Infliximab	Immune	None reported	
2	26 f	Crohn's	4 yrs	Calf	Infliximab	Immune	Mesalamine, GH	Severe Crohn's, registry azathioprine lansoprazole
3	39 m	Crohn's	2.5 yrs	Unknown	Infliximab	Immune	Mesalamine, Azathioprine	
4	f	MS	9-10 mos	Clavicle	Interferon	Immune	Potassium	pmh: clavicle fibrodysplasia, beta-1A, FMH positive for cancer
5	68 f	MS	>5 yrs	Shoulder	Interferon	Immune	No information	beta-1A
6	54 f	MS	3 mos	Brain	Natalizumab	immune	No information	
7	m	Ankylosing spondylitis	unknown	Unknown	Infliximab	Immune	No information	
8	50 f	RA	4 yrs	Unknown	Etanercept	Immune	No information	Canada
								
9	20 m	Psoriasis	6 yrs	Unknown	Infliximab	Immune	Methotrexate, cyclosporine, etanercept	Acitretin, efalizumab >6 yrs
10	52 m	transplant	8 yrs	Abdomen	Cyclosporine	Immune	steroids, azathioprine	Lliterature case [18] patient had OKT3 ab 2 transplants, nephrosclerosis
								
11	65 m	Chronic hepatitis C	12 yrs	Unknown	Peginterferon	Immune		Cholecystectomy, cirrhosis, esophageal varices alfa-2b and ribavirin
12	5 f	Nephrotic sx	18 mos	Spine	Cyclosporine	Immune	Dipyramidole, warfarin, prednisolone, cyclosporin	
13	11 f	GH def	18 mos	Thigh	r-somatropin	Hormone		Dose 0.7 mg sq should have been 0.47
14	16 f	GH def	Unknown	Clavicle	r-somatropin	Hormone		Patient grew 2.5 cm after GH
15	47 f	Menopause	10 mos	Spine	Estrogen/progestin	hormone	No information	Medical history—negative
16	24 f	Contraception	2.5 yrs	Unknown	Etonogestrel	Hormone	No information	Patient wt 268 lbs, ht 66.9′′, BMI = 42
17	49 f	Osteoporosis	4 mos	Shoulder	Teriparatide	Hormone		Other osteoporosis drugs, family history—breast cancer
18	17 m	Unspecified psychiatric condition	unknown	Unknown	Olanzapine	Psychotropic	Acetaminophen	
19	19 m	Depression	3 mos	Unknown	Olanzapine	Psychotropic	Clonazepam	Unspecified depression
20	8 m	Epilepsy	8 yrs	metastatic	Ethosuximide	Psychotropic	Clobazam, valproic acid	CNS depressant
21	8 f	ADHD	Unknown	Mnknown	Methylphenidate	Psychotropic		
22	34 f	Narcolepsy	22 mos	Unknown	Sodium oxybate	Psychotropic	None	
23	42 f	Sciatica	Unknown	Unknown	Pregabalin	Psychotropic	Lisinopril for high blood pressure	Fibromyalgia, CNS depressant peripheral neuropathy
24	24 f	Acne	10 mos	Unknown	Isotretinoin	Others	No information	
25	9 m	High cholesterol	23 mos	Mandible	Atorvastatin	Cholesterol Lowering	Ezetimibe	3-year trial

**Table 3:** Product labeling information on carcinogenicity for medications reported in FAERS that were used pre-ESFT diagnosis (Drugs@fda)^∗^.

Black box warning:	Adalimumab, azathioprine, cyclosporine, etanercept, everolimus, infliximab, teriparatide

Warning and precautions:	Recombinant human growth hormone or somatropin, methotrexate, estrogen/medroxyprogesterone

Cancer occurrence in mice or rat studies	Isotretinoin, olanzapine, atorvastatin, methylphenidate, pregabalin, valproic acid

No increase in tumor growth rates or metastasis in mouse xenograft transplant studies	Natalizumab

No carcinogenic risk detected in long-term studies:	Clozapine

No carcinogenic risk in animal studies conducted:	Mesalamine, etonogestrel implant, xyrem

No information on carcinogenicity in labeling:	Ethosuximide

Carcinogenicity testing not adequately addressed:	Clobazam

Carcinogenicity testing not done:	Interferon beta-1a, clonazepam, peginterferon alfa-2b

^∗^ http://www.accessdata.fda.gov/scripts/cder/drugsatfda/.

**Table 4:** ESFT-related studies.

Author/publication year/age group	Number of cases	Number of controls/cohort	Findings
Case-control studies			
Buckley et al., 1998 [10], (<21 yrs)	153	153	Inverse association with asthma. Looked at diseases and treatments, but not specifically medications. Earlier growth spurt and lower gain in weight and height among males but ES females no differences between cases and controls during growth spurt associations between GU anomalies and ES could not be confirmed.
Hartley et al., 1988 [9], (<15 yrs)	43 soft tissue & bone cancers (16 ES)	146	Developmental anomalies in 5 ES children: 1 meningomyelocele; 1 with an absent kidney and ureter. Medications were evaluated first month of life, 1–5 months, ≥6 months and grouped as antibiotics, anticonvulsants, corticosteroids, anti-allergic, bronchodilators, decongestants, cough suppressants and expectorants, and drugs for GI disorders.
Holly et al., 1992 [11], (<31 yrs)	43	193	Agricultural exposures, overdose of medications or accidental ingestion of poisonings
Winn et al., 1992 [12], (5 mos–22 yrs)	208	395	Hernias ~6 times more than expected, (OR 5.7; 95% Cl, 1.7–19.3) and also excess of cardiac conditions which however were mostly functional heart murmurs
Valery et al., 2003 [16], (most <20 yrs, ~75%)	106	344	Disorders of the digestive tract, behavioral hyperactivity and disorder of male organs (hydrocele and cryptorchidism) were also more frequent in cases but were not statistically significant. Only hernia excess achieved statistical significance (OR 3.1, 95% CI 1.2–7.6). Inverse association with asthma; deficit of bone disorders in cases (mostly fractures); also less frequent family history of stomach and neuroectodermal cancers. Authors state inverse association with medications: Use of medication and medical procedures steroids 9 cases, 30 controls 1.2 (0.5–2.9) anti-epileptic 1 case, 4 controls 0.9 (0.1–9.3) antibiotic 95 cases, 324 controls 0.6 (0.3–1.5) vermicide 62 cases, 246 controls 0.5 (0.3–0.8) hormone 6 cases, 51 controls 0.5 (0.2–1.5)

Cohort studies: case series or registries			
Pendergrass et al. 1984 [19], (≤18 yrs)	291	n/a	No strong association with stature
Cope et al. 2000 [13], (<46 yrs)	306	n/a	13 inguinal hernias, also 14 bony anomalies, 5 undescended testes, 1 abnormal kidney, 6 duplication of ureters, Zollinger-Ellison syndrome (which is associated with pituitary tumors)
Narod et al., 1997 [14], (<15),	396 ES (out of 20,304 cancers)	23 (5.8% anomalies)	National Registry Britain for Childhood Tumors (NRCT) and the BC British Registry for anomalies for presence of anomalies note in this study CNS PNETs were classified with medulloblastomas not part of ES. 2 cases osteogenic imperfecta. (confirmed previous associations that McKeen et al. [15] found)
McKeen et al., 1983 [15], (<46 yrs)	154 (23 cases ES)		Genitourinary, musculoskeletal. 56 development anomalies, 19 GU, of 99 males, 2 with unilateral cryptorchidism, 2 hypospadias, 5 of 55 females with unilateral ureter duplications, 8 rib anomalies, 7 vertebral defects, 4 with benign bone neoplasms (2 at primary ESFT site were bone cyst and enchondroma)
Glass and Fraumeni 1970 [20] (<15 yrs)	146 (out of 396 childhood cancers)		Hospital series, 2 spina bifida (1 with café au lait spots), osteoid osteoma, bone cysts, cryptorchidism, varicocele, Meckel's diverticulum, colonic polyps w/accessory spleen, congenital pulmonic stenosis, pulmonic valve 4 cusps, 1 mongolism (Down syndrome), 2 mothers of ES pts had multiple sclerosis; thyroidectomy for goiters in 2 mothers of ES pts.
Beyaert et al., 2013 [21]			Rib anomalies ES with high incidence of cervical ribs. 17.1% (not confirmed by other studies)

Pooled analysis and meta-analysis of studies regarding hernias in association with ESFT			
Valery et al. 2003 [16]	199 cases	1,451 controls	Association with hernias, umbilical, inguinal, and congenital. The primary endpoint was development of a tumor from the Ewing's sarcoma family. 138 patients with such a tumor and 574 controls were included in the pooled analysis, and 357 patients with these tumors and 745 controls were included in the meta-analysis.

## References

[1] Jawad M. U., Cheung M. C., Min E. S., Schneiderbauer M. M., Koniaris L. G., Scully S. P. (2009). Ewing sarcoma demonstrates racial disparities in incidence-related and sex-related differences in outcome: an analysis of 1631 cases from the SEER database, 1973–2005. *Cancer*.

[2] Burningham Z., Hashibe M., Spector L., Schiffman J. D. (2012). The epidemiology of sarcoma. *Clinical Sarcoma Research*.

[3] Ladanyi M. (2002). EWS-FLI1 and Ewing's sarcoma: recent molecular data and new insights. *Cancer Biology & Therapy*.

[4] Ambros I. M., Ambros P. F., Strehl S., Kovar H., Gadner H., Salzer-Kuntschik M. (1991). MIC2 is a specific marker for ewing's sarcoma and peripheral primitive neuroectodermal tumors. Evidence for a common histogenesis of ewing's sarcoma and peripheral primitive neuroectodermal tumors from MIC2 expression and specific chromosome aberration. *Cancer*.

[5] Javery O., Krajewski K., O'Regan K. (2011). A to Z of extraskeletal Ewing sarcoma family of tumors in adults: imaging features of primary disease, metastatic patterns, and treatment responses. *The American Journal of Roentgenology*.

[6] Esiashvili N., Goodman M., Marcus R. B. (2008). Changes in incidence and survival of ewing sarcoma patients over the past 3 decades: surveillance epidemiology and end results data. *Journal of Pediatric Hematology/Oncology*.

[7] Worch J., Matthay K. K., Neuhaus J., Goldsby R., Dubois S. G. (2010). Ethnic and racial differences in patients with ewing sarcoma. *Cancer*.

[8] Randall R. L., Lessnick S. L., Jones K. B. (2010). Is there a predisposition gene for ewing's sarcoma?. *Journal of Oncology*.

[9] Hartley A. L., Birch J. M., McKinney P. A. (1988). The Inter-Regional Epidemiological Study of Childhood Cancer (IRESCC): case control study of children with bone and soft tissue sarcomas. *British Journal of Cancer*.

[10] Buckley J. D., Pendergrass T. W., Buckley C. M. (1998). Epidemiology of osteosarcoma and Ewing's sarcoma in childhood: a study of 305 cases by the children's cancer group. *Cancer*.

[11] Holly E. A., Aston D. A., Ahn D. K., Kristiansen J. J. (1992). Ewing's bone sarcoma, paternal occupational exposure, and other factors. *American Journal of Epidemiology*.

[12] Winn D. M., Li F. P., Robison L. L., Mulvihill J. J., Daigle A. E., Fraumeni J. F. (1992). A case-control study of the etiology of Ewing's sarcoma. *Cancer Epidemiology Biomarkers and Prevention*.

[13] Cope J. U., Tsokos M., Helman L. J., Gridley G., Tucker M. A. (2000). Inguinal hernia in patients with Ewing sarcoma: a clue to etiology. *Medical and Pediatric Oncology*.

[14] Narod S. A., Hawkins M. M., Robertson C. M., Stiller C. A. (1997). Congenital anomalies and childhood cancer in Great Britain. *American Journal of Human Genetics*.

[15] McKeen E. A., Hanson M. R., Mulvihill J. J., Glaubiger D. L. (1983). Birth defects with Ewing's sarcoma. *The New England Journal of Medicine*.

[16] Valery P. C., McWhirter W., Sleigh A., Williams G., Bain C. (2003). A national case-control study of Ewing's sarcoma family of tumours in Australia. *International Journal of Cancer*.

[17] MedDRA http://www.meddra.org/.

[18] Balakrishnan R., Khairullah Q. T., Giraldo A., Provenzano R. (1999). Extraskeletal Ewing's sarcoma in a kidney transplant patient. *American Journal of Kidney Diseases*.

[19] Pendergrass T. W., Foulkes M. A., Robison L. L., Nesbit M. E. (1984). Stature and Ewing's sarcoma in childhood. *American Journal of Pediatric Hematology/Oncology*.

[20] Glass A. G., Fraumeni J. F. (1970). Epidemiology of bone cancer in children. *Journal of the National Cancer Institute*.

[21] Beyaert R., Beaugerie L., van Assche G. (2013). Cancer risk in immune-mediated inflammatory diseases (IMID). *Molecular Cancer*.

[22] Habel L. A., Friedman G. D., Schottenfeld D., Fraumeni J. F. (2006). Chapter 25. Phaamaceuticals other than hormones. *Textbook of Cancer Epidemiology and Prevention*.

[23] Engels E. A., Pfeiffer R. M., Fraumeni J. F. (2011). Spectrum of cancer risk among US solid organ transplant recipients. *Journal of the American Medical Association*.

[24] Opelz G., Henderson R. (1993). Incidence of non-Hodgkin lymphoma in kidney and heart transplant recipients. *The Lancet*.

[25] Hudesman D., Lichtiger S., Sands B. (2013). Risk of extraintestinal solid cancer with anti-TNF therapy in adults with inflammatory bowel disease: review of the literature. *Inflammatory Bowel Diseases*.

[26] Peyrin-Biroulet L., Deltenre P., de Suray N., Branche J., Sandborn W. J., Colombel J.-F. (2008). Efficacy and safety of tumor necrosis factor antagonists in Crohn’s disease: meta-analysis of placebo-controlled trials. *Clinical Gastroenterology and Hepatology*.

[27] Vial T., Descotes J. (2003). Immunosuppressive drugs and cancer. *Toxicology*.

[28] Penn I. (1995). Sarcomas in organ allograft recipients. *Transplantation*.

[29] Gridley G., McLaughlin J. K., Ekbom A. (1993). Incidence of cancer among patients with rheumatoid arthritis. *Journal of the National Cancer Institute*.

[30] Mellemkjær L., Linet M. S., Gridley G., Frisch M., Møller H., Olsen J. H. (1996). Rheumatoid arthritis and cancer risk. *European Journal of Cancer Part A*.

[31] Kinlen L. J. (1985). Incidence of cancer in rheumatoid arthritis and other disorders after immunosuppressive treatment. *The American Journal of Medicine*.

[32] Zintzaras E., Voulgarelis M., Moutsopoulos H. M. (2005). The risk of lymphoma development in autoimmune diseases: a meta-analysis. *Archives of Internal Medicine*.

[33] Lebrun C., Debouverie M., Vermersch P. (2008). Cancer risk and impact of disease-modifying treatments in patients with multiple sclerosis. *Multiple Sclerosis*.

[34] Nielsen N. M., Rostgaard K., Rasmussen S. (2006). Cancer risk among patients with multiple sclerosis: a population-based register study. *International Journal of Cancer*.

[35] Kingwell E., Tremlett H. (2009). Interferons and multiple sclerosis: is it plausible that *β*-IFN treatment could influence the risk of cancer among MS patients?. *Expert Review of Neurotherapeutics*.

[36] Darby S. C., Nakashima E., Kato H. (1985). A parallel analysis of cancer mortality among atomic bomb survivors and patients with ankylosing spondylitis given X-ray therapy. *Journal of the National Cancer Institute*.

[37] Weiss H. A., Darby S. C., Doll R. (1994). Cancer mortality following x-ray treatment for ankylosing spondylitis. *International Journal of Cancer*.

[38] Widhe B., Widhe T. (2000). Initial symptoms and clinical features in osteosarcoma and Ewing sarcoma. *The Journal of Bone & Joint Surgery—American Volume*.

[39] Frassica F. J., Frassica D. A., Pritchard D. J., Schomberg P. J., Wold L. E., Sim F. H. (1993). Ewing sarcoma of the pelvis. Clinicopathological features and treatment. *The Journal of Bone and Joint Surgery—American Volume*.

[40] Damron T. A., Sim F. H., O'Connor M. I., Pritchard D. J., Smithson W. A. (1996). Ewing's sarcoma of the proximal femur. *Clinical Orthopaedics & Related Research*.

[41] Sundaram M., Inwards C. Y., Shives T. E., Anderson P. M. (2005). Ewing's sarcoma of the humerus mimicking fibrous dysplasia on imaging and biological behavior. *Skeletal Radiology*.

[42] Kato K., Hayashi T., Tabuchi K. (2007). Concurrent Ewing sarcoma family of tumors and fibrous dysplasia: possible diagnostic pitfall. *Journal of Pediatric Hematology/Oncology*.

[43] Jacobs A., Jacobson-Kram D. (2004). Human carcinogenic risk evaluation, part III: assessing cancer hazard and risk in human drug development. *Toxicological Sciences*.

[44] Bugelski P. J., Volk A., Walker M. R., Krayer J. H., Martin P., Descotes J. (2010). Critical review of preclinical approaches to evaluate the potential of immunosuppressive drugs to influence human neoplasia. *International Journal of Toxicology*.

[45] Gaona-Luviano P., Unda-Franco E., González-Jara L., Romero P., Medina-Franco H. (2003). Primitive neuroectodermal tumor of the vagina. *Gynecologic Oncology*.

[46] Peres E., Mattoo T. K., Poulik J., Warrier I. (2005). Primitive neuroectodermal tumor (PNET) of the uterus in a renal allograft patient: a case report. *Pediatric Blood and Cancer*.

[47] Penn I. (2000). Post-transplant malignancy: the role of immunosuppression. *Drug Safety*.

[48] Ruangkanchanasetr P., Lauhawatana B., Leawseng S., Kitpanich S., Lumpaopong A., Thirakhupt P. (2012). Malignancy in renal transplant recipients: a single-center experience in Thailand. *Journal of the Medical Association of Thailand*.

[49] Carel J.-C., Ecosse E., Landier F. (2012). Long-term mortality after recombinant growth hormone treatment for isolated growth hormone deficiency or childhood short stature: preliminary report of the French SAGhE study. *The Journal of Clinical Endocrinology & Metabolism*.

[50] Banerjee I., Clayton P. E. (2007). Growth hormone treatment and cancer risk. *Endocrinology and Metabolism Clinics of North America*.

[51] Bell J., Parker K. L., Swinford R. D., Hoffman A. R., Maneatis T., Lippe B. (2010). Long-term safety of recombinant human growth hormone in children. *Journal of Clinical Endocrinology and Metabolism*.

[52] Sävendahl L., Maes M., Albertsson-Wikland K. (2012). Long-term mortality and causes of death in isolated GHD, ISS, and SGA patients treated with recombinant growth hormone during childhood in Belgium, The Netherlands, and Sweden: preliminary report of 3 countries participating in the EU SAGhE study. *The Journal of Clinical Endocrinology & Metabolism*.

[53] Ogilvy-Stuart A. L., Gleeson H. (2004). Cancer risk following growth hormone use in childhood: implications for current practice. *Drug Safety*.

[54] Nishi Y., Tanaka T., Takano K. (1999). Recent status in the occurrence of leukemia in growth hormone-treated patients in Japan. *The Journal of Clinical Endocrinology & Metabolism*.

[55] Sklar C. A., Mertens A. C., Mitby P. (2002). Risk of disease recurrence and second neoplasms in survivors of childhood cancer treated with growth hormone: a report from the childhood cancer survivor study. *Journal of Clinical Endocrinology and Metabolism*.

[56] Mo D., Hardin D. S., Erfurth E. M., Melmed S. (2014). Adult mortality or morbidity is not increased in childhood-onset growth hormone deficient patients who received pediatric GH treatment: an analysis of the Hypopituitary Control and Complications Study (HypoCCS). *Pituitary*.

[57] Chemaitilly W., Robison L. L. (2012). Safety of growth hormone treatment in patients previously treated for cance. *Endocrinology and Metabolism Clinics of North America*.

[58] Hagay Z. J., Leiberman R. J., Katz M., Witznitzer A. (1988). Oral contraceptives and focal nodular hyperplasia of the liver. *Archives of Gynecology and Obstetrics*.

[59] Valesini G., Cutolo M. (2005). Effects of TNF antagonists on immune and neuroendocrine system. *Reumatismo*.

[60] Madhusoodanan S., Parida S., Jimenez C. (2010). Hyperprolactinemia associated with psychotropics—a review. *Human Psychopharmacology*.

[61] Corell C. U., Carlson H. E. (2006). Endocrine and metabolic adverse effects of psychotropic medications in children and adolescents. *Journal of the American Academy of Child and Adolescent Psychiatry*.

[62] Herzog A. G., Fowler K. M. (2005). Sexual hormones and epilepsy: threat and opportunities. *Current Opinion in Neurology*.

[63] Karadag A. S., Ertugrul D. T., Tutal E., Akin K. O. (2011). Isotretinoin influences pituitary hormone levels in acne patients. *Acta Dermato-Venereologica*.

[64] Bilo L., Meo R., Valentino R., Di Carlo C., Striano S., Nappi C. (2001). Characterization of reproductive endocrine disorders in women with epilepsy. *The Journal of Clinical Endocrinology & Metabolism*.

[65] Jacobsen N. W., Halling-Sørensen B., Birkved F. K. (2008). Inhibition of human aromatase complex (CYP19) by antiepileptic drugs. *Toxicology in Vitro*.

[66] Fond G., Macgregor A., Attal J. (2012). Antipsychotic drugs: pro-cancer or anti-cancer? A systematic review. *Medical Hypotheses*.

[67] Nielsen J., Boysen A. (2010). Clozapine treatment associated with increased risk of acute myeloid leukemia (AML). *Schizophrenia Research*.

[68] Valery P. C., Holly E. A., Sleigh A. C., Williams G., Kreiger N., Bain C. (2005). Hernias and Ewing's sarcoma family of tumours: a pooled analysis and meta-analysis. *The Lancet Oncology*.

[69] Schumacher R., Mai A., Gutjahr P. (1992). Association of rib anomalies and malignancy in childhood. *European Journal of Pediatrics*.

[70] Miller R. W. (1969). Childhood cancer and congenital defects. A study of U.S. death certificates during the period 1960–1966. *Pediatric Research*.

[71] Casorzo L., Fessia L., Sapino A., Ponzio G., Bussolati G. (1989). Extraskeletal Ewing's tumor with translocation t(11;22) in a patient with Down syndrome. *Cancer Genetics and Cytogenetics*.

[72] Bridge J. A., Neff J. R., Borek D. A., Hackbarth D. A. (1990). Primary skeletal Ewing's sarcoma in Down syndrome. *Cancer Genetics and Cytogenetics*.

[73] Machin Valtueña M., Garcia-Sagredo J. M., Muñoz Villa A., Lozano Giménez C., Aparicio Meix J. M. (1987). 18q-syndrome and extraskeletal Ewing's sarcoma. *Journal of Medical Genetics*.

[74] Cotterill S. J., Wright C. M., Pearce M. S., Craft A. W. (2004). Stature of young people with malignant bone tumors. *Pediatric Blood and Cancer*.

